# Performance Evaluation of Heart Sound Cancellation in FPGA Hardware Implementation for Electronic Stethoscope

**DOI:** 10.1155/2014/587238

**Published:** 2014-03-25

**Authors:** Chun-Tang Chao, Nopadon Maneetien, Chi-Jo Wang, Juing-Shian Chiou

**Affiliations:** Department of Electrical Engineering, Southern Taiwan University of Science and Technology, No. 1 Nan-Tai Street, Yong Kung District, Tainan 71005, Taiwan

## Abstract

This paper presents the design and evaluation of the hardware circuit for electronic stethoscopes with heart sound cancellation capabilities using field programmable gate arrays (FPGAs). The adaptive line enhancer (ALE) was adopted as the filtering methodology to reduce heart sound attributes from the breath sounds obtained via the electronic stethoscope pickup. FPGAs were utilized to implement the ALE functions in hardware to achieve near real-time breath sound processing. We believe that such an implementation is unprecedented and crucial toward a truly useful, standalone medical device in outpatient clinic settings. The implementation evaluation with one Altera cyclone II–EP2C70F89 shows that the proposed ALE used 45% resources of the chip. Experiments with the proposed prototype were made using DE2-70 emulation board with recorded body signals obtained from online medical archives. Clear suppressions were observed in our experiments from both the frequency domain and time domain perspectives.

## 1. Introduction

Significant advances on health care, especially on medical devices, have been made in the past few decades. However, albeit basic, stethoscope is still essential for the diagnoses of many diseases. In fact, the electronic stethoscope is one medical instrument studied recently by many groups of researchers [1–9]. Numerous new features were studied, and some of them are made commercially available [10, 11]. Representative issues extensively studied on electronic stethoscope are background noise reduction, heart sound enhancement, telecommunication capabilities, and recording features to aid diagnosis.

The preliminary diagnosis of pulmonary diseases in outpatient clinics hinges on the identification of abnormal audible features in breath sounds such as crackles and wheezes by using stethoscope. These “adventitious breath sounds” are superimposed over normal ones. Also, there is an intrinsic spectral overlap of heart sounds and lung sounds. This means that heart sounds, when unintentionally received by the stethoscope pickup due to the proximity of the heart and areas of stethoscope lung examination, may interfere with the identification of adventitious breath sounds. It would then be helpful for diagnosis, especially in the outpatient clinic settings, if electronics can be employed to attenuate the audio components resulting from the heart beat in a near real-time manner.

Some studies looked into the separation of heart sounds and lung sounds [12–22] of recorded body sounds via a personal computer in an offline fashion. To the best of our knowledge, none of these works discussed the realization of a hardware-based device capable of performing near real-time heart sound denoising from lung sound signals; also, all the experiment results shown in these works were simulations in software.

An adaptive filter with “Augmented ECG” as a reference signal was applied to filter out heart sounds from lung sounds in Iyer et al. [12]. In [13], two FIR adaptive filters with the delay reference signal were used to generate the first and second heart sounds, respectively. Yip and Zhang proposed a new stethoscope design with Laplacian ECG (LECG) as a reference signal and developed an algorithm to separate heart and lung sounds. This algorithm was implemented on a LabVIEW platform with an IBM-compatible PC [14]. ALE has been proposed in [15] to separate a heart sound signal from wheezy lung sound signal, or the term “colored noise” used therein [15] also indicated that the performance depends strongly on the choice of delay. In [16], fourth-order statistics of the entire recorded signal were incorporated in an adaptive filtering structure to reduce heart sounds from lung sounds. A “heart sound tracking algorithm” generates the reference input for the adaptive filter. Wavelet-based methods to reduce heart sound noise from lung sounds were proposed by Hadjileontiadis and Panas in [17] and Hossain and Moussavi in [18]. Using time-frequency filtering to cancel heart sound from lung sound has been proposed in [19]. Independent component analysis was adopted for the separation of heart from lung sound in [20, 21]. The recently proposed methodology to reduce heart sound noise from lung is empirical mode decomposition (EMD) technique [22].

As we mentioned, a convenient setup “extracting” lung sounds in real time has never been developed. In this paper, we propose the design of hardware circuit capable of attenuating heart sound attributes from lung sound signal based on FPGAs for electronic stethoscope. This will be helpful for physicians or pulmonary specialist to find out problems and save patient's life as soon as possible.

## 2. Materials and Methods

### 2.1. Prototype of FPGA Hardware Implementation with ALE

In the literature, electronic stethoscope had been implemented by using general-purpose microcontrollers (MCUs) [2–5, 9], DPS chips [1, 7, 8], or even ASIC chips [6]. We chose to build the proposed prototype of ALE circuit using FPGAs instead. In general, algorithms like adaptive filtering or adaptive noise cancellation cannot be implemented in most MCUs because the computational requirements are beyond their specifications. We favor FPGAs over DSPs for their flexibility of parallel hardware realization which is the most economical for adaptive filtering. ASIC design generally requires a high budget and is not practical for prototype development. Unlikely the MUCs, DSPs and FPGAs are the devices with numerous logic gates and RAM blocks that are flexible to be implemented in either pure hardware (All logic gate), customer Soft CPU (e.g., NIOS II Processor from Altera or MicroBlaze from Xilinx), or hardware/software codesign to enhance the speed and reduce the complexity in software programming. In this prototype, we used pure hardware implementation by using VHDL (VHSIC hardware description language) to describe the hardware architecture in FPGA. FPGAs have also been used in many applications such as wireless sensors network (WSN) [23], pulse oximeter [24], adaptive filters reducing power line noise in ECG measurement system [25], and EMG measurement power line noise cancellation [26].

**Figure 1:**
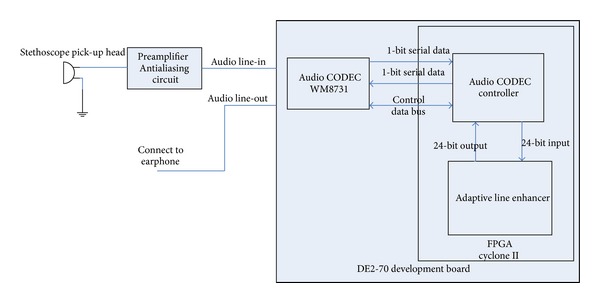
Proposed prototype block diagram.

The proposed prototype consists of 2 main function units. The first one is adaptive line enhancer (ALE), which will be described in Section 2.2. The other is the audio CODEC controller. DE2-70 Board features an audio CODEC using WM8731 chip running at low power and includes a high efficient headphone driver, which is very useful for the proposed electronic stethoscope project. The WM8731 can be controled via 2 or 3 wire serial interfaces to set ADC and DAC sampling rates, audio data interface modes (I2S, left, right justified or DSP), and word lengths (16/20/24/32 bit). In this work, ADC/DAC sampling rates are 48 kHz with 24-bit wordlength and the left justified mode is selected. The block diagram of the electronic stethoscope prototype is shown in Figure 1. The signal from audio line-in terminal of DE2-70 was first passed through audio CODEC WM8731 with 24-bit serial output. The CODEC controller module further converts the serial data to parallel ones for the simplicity of downstream processing.

### 2.2. Algorithm/Model

Since Widrow et al. first introduced the adaptive filter to suppress noise from desired signal in 1975 [27, 28], adaptive filters have been developed to include many applications. Normally, adaptive filter can be used to reduce heart sound noise from lung sound as in [12, 14], but, for electronic stethoscope to be used in outpatient clinic settings, it is not convenient because adaptive filter requires an external reference signal. In this work, we choose ALE instead.

**Figure 2:**
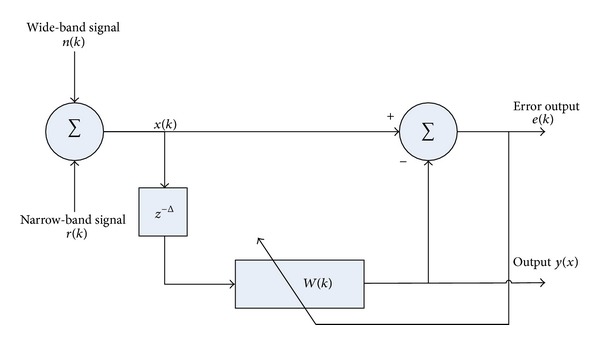
Basic structure of ALE.

Figure 2 presents the ALE basic structure. It consists of an *L*-weight linear prediction FIR filter and a variable delay of input signal. The filter weight can be adjusted by least mean square (LMS) adaption algorithm. As shown in Figure 2, *x*(*k*) consists of a narrow-band signal *r*(*k*) and a board band signal *n*(*k*):

(1) $$\begin{array}{c} x\left(k\right)=r\left(k\right)+n\left(k\right). \end{array}$$

In our case, *x*(*k*) is the signal we acquired from microphone connected to stethoscope pick-up head, which is also the combination of heart sound *r*(*k*), a narrow-band signal, and lung sound *n*(*k*), a broad band signal.

The output *y*(*k*) of ALE is defined as

(2) $$\begin{array}{c} y\left(k\right)=\overset{L-1}{\underset{l=0}{\sum }}w_{l}\left(k\right)x\left(k-l-\Delta \right), \end{array}$$

where Δ is the prediction distance of the filter in terms of the sampling period, *L* is the filter length, and *w*_*l*_(*k*) is the FIR filter weight (ALE coefficients).

The least mean square (LMS) algorithm is a widely used algorithm for adaptive filtering. It is based on the approximation of the gradient toward the optimal point using statistical properties of the input signal. The filter weights adapt in response to the error for each coming new sample. To adjust the ALE coefficients, the LMS algorithm is preferable for its computational simplicity and robustness. The adaption is described by

(3) $$\begin{array}{c} W\left(k+1\right)=W\left(k\right)+\mu X\left(k-\Delta \right)\left(x\left(k\right)-y\left(k\right)\right), \end{array}$$

where *W*(*k*) = [*w*_0_(*k*),…,*w*_*L*−1_(*k*)]^*T*^ in which *L* is adaptive filter length, *X*(*k* − Δ) = [*x*(*k* − Δ),…, *x*(*k* − Δ − *L* + 1)] is the input vector, and *μ* is the convergence parameter.

Three important parameters in LMS algorithm, adaptive filter length *L*, the prediction distance Δ, and the convergence parameter *μ*, will affect the performance ALE in terms of adaption rate, excess mean squared error (EMSE), and frequency resolution. The adaptation rate is controlled by selection of *μ* and *L* and the condition of data vector autocorrelation.

According to Figure 2, the input signal *x*(*k*) in the intended scenario of usage of our proposed stethoscope is the body sound from the stethoscope pickup consisting of heart sound and lung sound. Since heart sound is a narrow-band quasiperiodic signal *r*(*k*) and lung sound is a wide-band signal *n*(*k*), the system output *y*(*k*) of proposed system should be the error output *e*(*k*).

**Figure 3:**
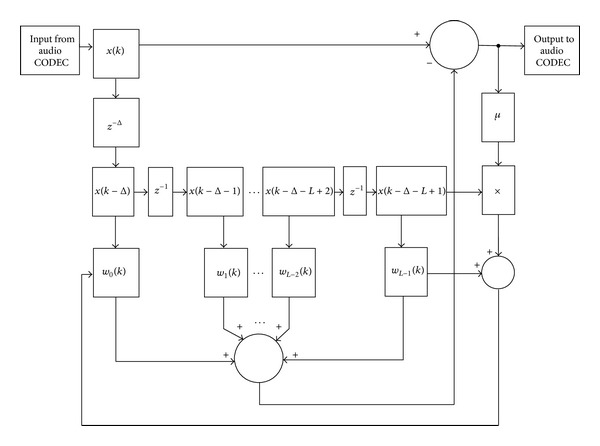
Hardware structure of ALE.

From basic structure of ALE in Figure 2 can implement in FPGA by use the hardware structure as shown in Figure 3. In Figure 3 show the consist of ALE hardware structure including FIR filter with *L* length, prediction distance *D* and convergence parameter *m*. The input of ALE are from audio CODEC and after processing ALE output will send to audio CODEC again to convert digital data to audio signal.

Three important parameters to design and select coding in VHDL have been chosen with the help of preliminary simulations carried out with MATLAB. We used a 10-tap filter, *μ* = 0.0001, and Δ = 32 for this prototype setup.

**Figure 4:**
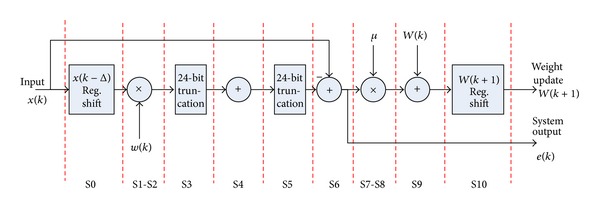
ALE computation steps.

Here, we need to check whether our hardware is indeed fast enough. Because we selected parallel hardware implementation to realize ALE, only 11 steps are necessary for each ALE adaptation as shown in Figure 4. This can be completed within 0.22 *μ*s at the 50 MHz system clock rate, well within one sampling period, or 20.8 *μ*s at 48 KHz.

## 3. Results and Discussion

**Figure 5:**
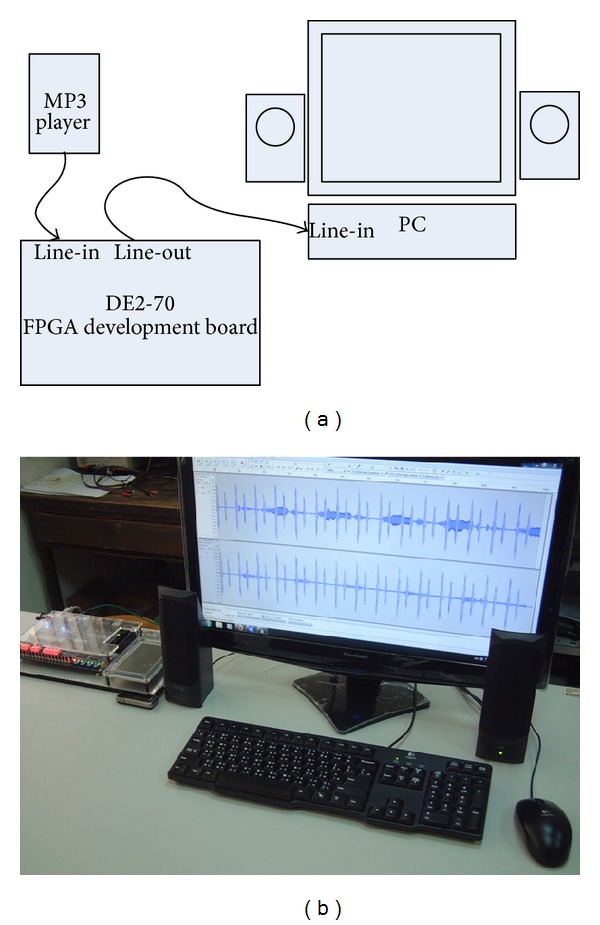
(a) Schematic diagram of experiment setup. (b) Photo of experiment setup.

The heart sound cancellation feature was evaluated based on the performance of electronic stethoscope with the ALE being implemented in FPGA with DE2-70 board as mentioned above. Two experiments were conducted. We first acquired original crackles and wheezes signal in MP3 file format from School of Medicine, University of Washington [29], and converted into WAV file format them to be easy to analyze in used program. Attributes of heart sounds were noticeable for both signals when played back. In this experiment, the signals were played back using an MP3 player with the analog output sent to the line-in input terminal of the DE2-70 board. After the processing of ALE, the analog output was sent to the line-in terminal of a personal computer. Subsequently, we used audacity for recording and then generated the spectrograms by using Sonic Visualiser [30]. The experiments setup is shown in Figure 5.

### 3.1. Spectrogram Result

**Figure 6:**
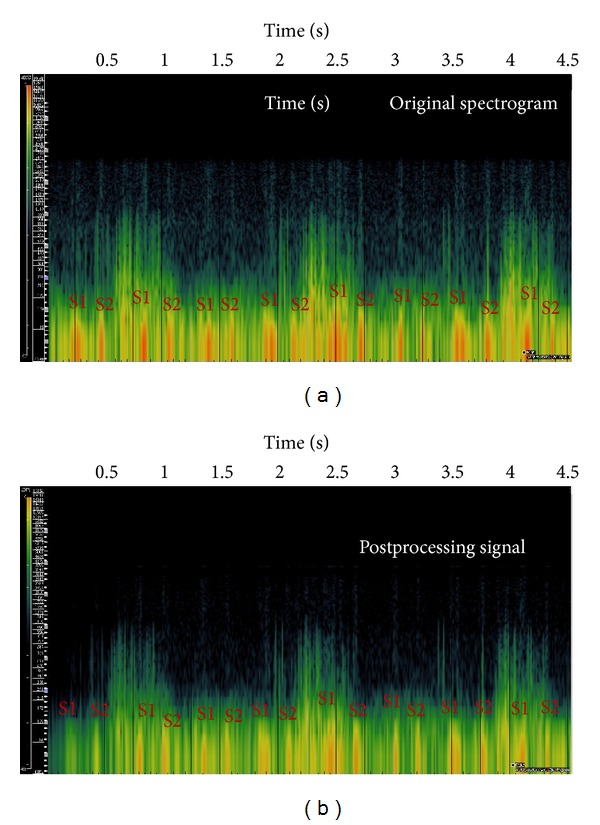
(a) The spectrogram of crackles with audible heart sounds. (b) The spectrogram after processing.

**Figure 7:**
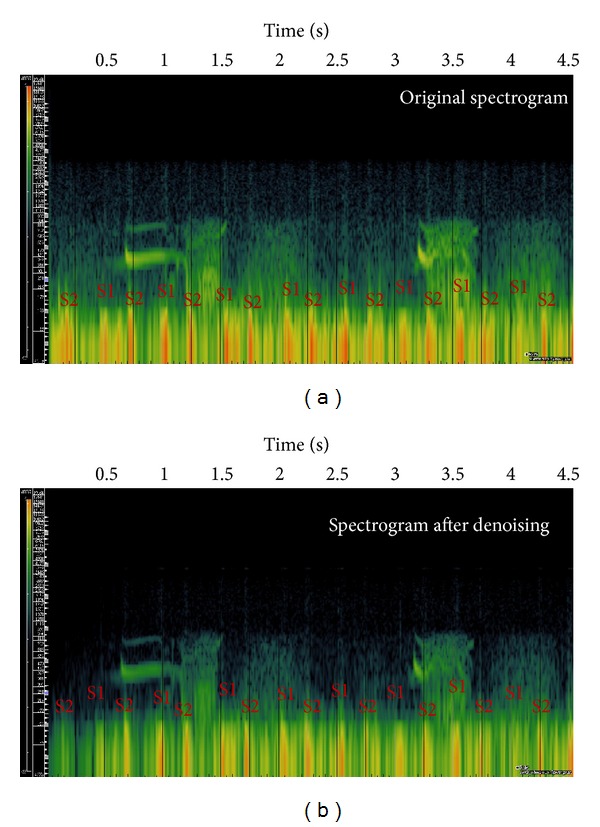
(a) The spectrogram of wheezes with audible heart sounds. (b) Spectrogram after processing.

The spectrograms of crackles before and after the processing are shown in Figure 6. Those of the wheezes are shown in Figure 7. The effectiveness of our proposed setup can be observed from the fact that the pulse-train-like signatures from heart sounds were clearly lessened in the spectrogram. S1 and S2 represent the first and second heart sounds, respectively.

### 3.2. Waveform Result

**Figure 8:**
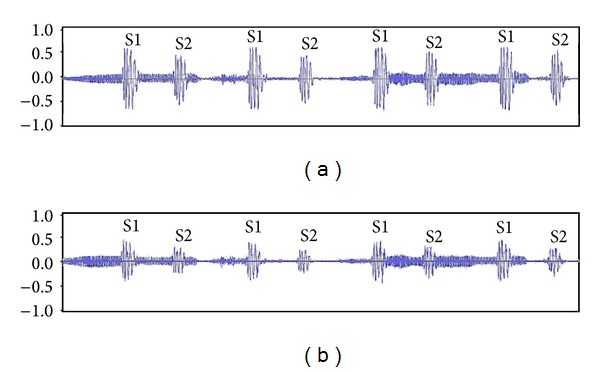
(a) The waveform of heart sounds mixed with wheezes. (b) The waveform after processing.

The second experiment aims to demonstrate the efficacy using time waveforms. We acquired the signals of heart sound and wheezes from [11]. We consider this signal to contain, very little if any, attributes from the heart sounds when recorded. The heart sounds and wheezes were intentionally mixed together with the ratio of gains for heart sounds to that for wheezes being 0.7 : 1. Please note that, in pulmonary examinations, physicians seek adventitious lung sounds, not heart sounds. This ratio would have been larger than the actual ones detected in different chest locations during lung sound auscultation. We then feed the mixed signal to ALE and followed similar procedures as those in experiment one. The time signals before and after processing are shown in Figure 8, where the attenuation of heart sounds can be apparently observed from the time waveforms.

### 3.3. Resources Usage

In Table 1, we list the gate resource usage for the 10-tap ALE and audio CODEC controller on target device EP2C70F89 with 68416 logic cells. As shown in this table, 10-tap ALE, the core of electronic stethoscope, can be implemented in FPGAs with parallel implementation at about 45% with high throughput.

Finally, we need to ensure that our proposed prototype is suitable for outpatient pulmonary examination. Specifically, we need to show that there will be no perceivable time lags in the proposed stethoscope. Note that, upon the application of stethoscope diaphragm, the inherent delay (*z* ^−Δ^) of ALE will result in an initial delay of 33 sampling periods, or roughly 0.7 ms for signals sampled at 48 KHz, before sending out audio signals to the headset. Such a time lag is by no means perceivable to humans.

## 4. Conclusions

In this paper, we presented the design and testing for an unprecedented ALE heart sound cancellation circuit developed on the FPGA DE2-70 education board for electronic stethoscopes. Our experiments show clear reduction of heart sounds from breath sounds; moreover, the electronic processing will not result in any perceivable delay that may discourage physicians from using this stethoscope. We believe the proposed heart sound cancellation hardware circuit for electronic stethoscope will be most useful in pulmonary examinations as well as medical education. In the near future, the implemented prototype will be evaluated in a clinical setting by medical doctors in the pulmonology department. A draft of application for institutional review board approval of a clinical trial is in preparation.

## Figures and Tables

**Table 1:** FPGA resource usage.

Function	Total number of logic elements	Total number of combination function	Total number of registers	Total number of embedded 9-bit multipliers
Audio CODEC controller	624 (<1%)	624 (<1%)	350 (<1%)	0 (0%)
10-tap ALE	2479 (4%)	2238 (3%)	1410 (2%)	135 (45%)
